# Human NOD2 Recognizes Structurally Unique Muramyl Dipeptides from Mycobacterium leprae

**DOI:** 10.1128/IAI.00334-16

**Published:** 2016-08-19

**Authors:** Mirjam Schenk, Sebabrata Mahapatra, Phuonganh Le, Hee Jin Kim, Aaron W. Choi, Patrick J. Brennan, John T. Belisle, Robert L. Modlin

**Affiliations:** a Division of Dermatology, Department of Medicine, David Geffen School of Medicine at University of California, Los Angeles, California, USA; b Department of Microbiology, Immunology and Molecular Genetics, David Geffen School of Medicine at University of California, Los Angeles, California, USA; c Mycobacteria Research Laboratories, Department of Microbiology, Immunology and Pathology, Colorado State University, Fort Collins, Colorado, USA; Weill Cornell Medical College

## Abstract

The innate immune system recognizes microbial pathogens via pattern recognition receptors. One such receptor, NOD2, via recognition of muramyl dipeptide (MDP), triggers a distinct network of innate immune responses, including the production of interleukin-32 (IL-32), which leads to the differentiation of monocytes into dendritic cells (DC). NOD2 has been implicated in the pathogenesis of human leprosy, yet it is not clear whether Mycobacterium leprae, which has a distinct MDP structure, can activate this pathway. We investigated the effect of MDP structure on the innate immune response, finding that infection of monocytes with M. leprae induces IL-32 and DC differentiation in a NOD2-dependent manner. The presence of the proximal l-Ala instead of Gly in the common configuration of the peptide side chain of M. leprae did not affect recognition by NOD2 or cytokine production. Furthermore, amidation of the d-Glu residue did not alter NOD2 activation. These data provide experimental evidence that NOD2 recognizes naturally occurring structural variants of MDP.

## INTRODUCTION

The ability of the innate immune response to defend against microbial invaders involves germ line-encoded pattern recognition receptors (PRRs) which detect highly conserved pathogen-associated molecular patterns (PAMPs) of infectious agents. One such PRR, nucleotide-binding oligomerization domain 2 (NOD2), is a cytoplasmic receptor belonging to the NOD-like receptor family. NOD2 recognizes muramyl dipeptide (MDP), part of the peptidoglycan (PG) cell walls of Gram-positive and Gram-negative bacteria (1, 2).

We previously found that activation of NOD2, but not Toll-like receptor 2/1 (TLR2/1) or NOD1, induced the production of interleukin-32 (IL-32) in human monocytes. In addition, NOD2 activation induced the IL-32-dependent differentiation of monocytes into dendritic cells (DC). These IL-32-derived DC were distinguished from granulocyte-macrophage colony-stimulating factor (GM-CSF)-differentiated DC in having the capacity to cross-present exogenous antigen via major histocompatibility complex (MHC) class I to CD8^+^ T cells (3). Cross-presentation facilitates the induction of CD8^+^ cytotoxic T cell responses against intracellular pathogens that reside in the endosomal pathway. The biological relevance of this pathway was demonstrated in leprosy, in which NOD2, IL-32, and CD1^+^ DC all were more highly expressed in skin lesions from patients with the self-limited tuberculoid (T-lep) form versus the progressive lepromatous leprosy (L-lep) form. The relevance of NOD2 in the pathogenesis of leprosy is further suggested by the association of NOD2 gene polymorphisms with susceptibility to leprosy (4, 5).

MDP is the minimal essential structure of bacterial peptidoglycan required for its immunological effects, including the activity of Freund's complete adjuvant, which contains mycobacterial cell walls (6, 7). Typically, immunologic studies of NOD2 activation are performed using a synthetic MDP analogue, characterized by *N*-acetylmuramyl-l-alanyl-d-isoglutamine (8), which is present in most bacteria. The MDP in Mycobacterium spp. has several structurally distinct features, based on studies with the readily cultivable M. smegmatis and M. tuberculosis. MDP in these mycobacteria contains muramic acid residues that are *N*-glycolylated as well as *N*-acetylated (9, 10). The carboxyl functions of the peptide side chains are also partially modified by amidation, methylation, or an additional Gly residue (11, 12).

MDP from *in vivo*-derived and noncultivable M. leprae possesses the basic structural features of MDP from other mycobacteria but with the replacement of the proximal l-alanine by glycine in the peptide side chain and the lack of *N*-glycolylated muramic acid residues (10, 13). Therefore, the M. leprae peptidoglycan-derived MDP is structurally unique compared to other mycobacteria, as well as other pathogenic bacteria, yet its ability to activate the innate immune response is unknown. Given that structural modifications of MDP can alter biological activity (8, 14–17), we investigated the relationship of the structure of the M. leprae MDP with its ability to trigger immune activation of human monocytes.

## MATERIALS AND METHODS

### Bacteria and microbial ligands.

For activation of monocytes and HEK-NOD2 reporter cells, we used MDP (1 μg/ml; Invivogen, San Diego, CA), LL-MDP (1 μg/ml; Invivogen, San Diego, CA), live M. leprae (multiplicity of infection [MOI] of 10), and sonicated M. leprae (10 μg/ml). M. leprae was obtained from the footpad of nu/nu mice as described previously (18) and was provided by James L. Krahenbuhl of the National Hansen's Disease Programs, Health Resources Service Administration, Baton Rouge, LA. The M. leprae fractions (M. leprae sonicate, mAGP, and peptidoglycan) were obtained from armadillo spleen-derived M. leprae and generated as described elsewhere (13, 19, 20). All reagents were tested for endotoxin by LAL assay (Limulus amebocyte lysate; detection limit, <0.1 EU/ml; Lonza, Anaheim, CA).

### Preparation and analysis of soluble muropeptide from M. leprae PG.

Peptidoglycan was isolated, solubilized, and fractionated by size exclusion chromatography on a Superdex peptide 10/300 GL column (Amersham Biosciences, Pittsburgh, PA) using the conditions described by Mahapatra et al. (10). The muropeptide-containing fractions were analyzed by liquid chromatography-mass spectrometry (LC-MS) using an Agilent 1200 series high-performance liquid chromatography (HPLC) system connected to a 6520 series accurate time-of-flight mass spectrometer (Q-TOF) by following conditions previously described (21). The positive ion-MS data were processed with Agilent MassHunter qualitative analysis software to identify potential compounds ions, followed by a search against a custom database containing calculated monoisotopic ion masses of possible uncross-linked and cross-linked muropeptides from M. leprae PG with a maximum molecular mass of 2 kDa to predict the structure of the compound ions. The amino acid compositions of the muropeptide fractionated by size exclusion chromatography were also analyzed by an EZ:faast GC-MS kit by following the manufacturer's instructions (Phenomenex, Torrance, CA). The concentrations of the muropeptides were normalized based on the abundance of diaminopimelic acid residues.

### Enzymatic synthesis of M. leprae MDP analogues.

UDP-*N*-acetylmuramic acid (UDP-MurNAc) was synthesized by following methods described previously (22). UDP-*N*-acetylmuramyl-glycinyl–d-glutamate was synthesized from UDP-MurNac, Gly, and d-Glu using recombinant MurC and MurD enzymes of Escherichia coli. The reaction buffer and conditions used have been described previously (23, 24). UDP-*N*-acetylmuramyl-glycinyl-d-isoglutamine was synthesized in a similar reaction, except d-Glu was replaced with d-isoglutamine. The reaction mixtures were deproteinated and the nucleotide-linked MDPs were purified by ion-exchange chromatography by following the methods described previously (23). Nucleotides were removed by hydrolysis in 0.2 M trifluoroacetic acid at 60°C for 1 h, and the resulting MurNAC-glycinyl–d-glutamate or MurNAC-glycinyl-d–isoglutamine was dried under a stream of N_2_, resuspended in water, purified by size exclusion chromatography, and then analyzed by LC-MS as described above. The amino acid compositions of MDPs were analyzed by GC-MS as described above.

### Monocyte purification.

We obtained whole blood from healthy donors (UCLA I.R.B. 11-001274) with informed consent. Peripheral blood mononuclear cells (PBMCs) were isolated using Ficoll (GE Healthcare, Pittsburgh, PA) gradient centrifugation, and monocytes were further enriched using a Percoll density gradient (GE Healthcare, Pittsburgh, PA) and subsequent adherence in 1% fetal calf serum (FCS) for 2 h or were purified using an EasySep human monocyte enrichment kit without CD16 depletion (Stemcell Technologies, Vancouver, Canada). Monocyte purity was found to be >80% as measured by CD14 expression. Cells were cultured for 24 h in RPMI with 10% FCS (Omega Scientific, Tarzana, CA), penicillin (50 U/ml), streptomycin (50 μg/ml), and sodium pyruvate (1 mM).

### Flow cytometry.

Cell surface expression of antigenic determinants was measured using epitope-specific antibodies, and cells were acquired and analyzed as described previously (25). For detection of CD1b, a monoclonal primary antibody (Bcd3.1; ATCC, Manassas, VA) was used, followed by an IgG1-specific secondary antibody (Invitrogen, Carlsbad, CA). Samples were acquired on an LSR II machine (BD, San Jose, CA) and analyzed using FlowJo software.

### Cytokine ELISAs.

Secreted IL-32 protein in the supernatant was measured using an IL-32 sandwich enzyme-linked immunosorbent assay (ELISA) kit (SEL101; YbdY Biotech, South Korea) or a matched antibody pair (BioLegend, San Diego, CA). To measure secreted IL-1β, IL-6, and tumor necrosis factor alpha (TNF-α), matched antibody pairs were used according to the manufacturer's recommendations (Biosource, San Diego, CA). For detection we used streptavidin-horseradish peroxidase (HRP) (1:1,000; Pierce, Rockford, IL) and ABTS [2,2′-azinobis(3-ethylbenzthiazolinesulfonic acid)] HRP substrate mixture (Kirkegaard and Perry Laboratories, Inc., Gaithersburg, MD), and plates were read at 405 nm.

### Real-time qPCR.

Following stimulation of monocytes, RNA was isolated using TRIzol (Invitrogen, Carlsbad, CA), cDNA and quantitative PCR (qPCR) was performed as previously described (26). QuantiTect primers (Qiagen, Hilden, Germany) were used. The relative quantities of the gene tested per sample were calculated against h36B4 using the delta cycle threshold formula as previously described (27). The data were normalized by fold change to medium control samples.

### HEK NOD2 reporter assay.

HEK-Blue hNOD2 reporter cells (Invivogen, San Diego, CA) were cultured in Dulbecco's modified Eagle's medium (DMEM), 4.5 g/liter glucose, 10% FCS (Omega Scientific, Tarzana, CA) with 50 U/ml penicillin, 50 μg/ml streptomycin, and 100 μg/ml normocin. For antibiotic selection, 30 μg/ml blasticidin and 100 μg/ml zeocin were added. HEK-Blue detection was achieved by adding 2.5 × 10^4^ cells to a 20-μl sample, which was incubated at 37°C in 5% CO_2_ for 16 h. Secreted embryonic alkaline phosphatase (SEAP) was quantified using HEK-Blue detection (Invivogen, San Diego, CA) and measured by a spectrophotometer at 655 nm.

## RESULTS

### M. leprae infection of human monocytes induces IL-32 via NOD2.

**FIG 1:**
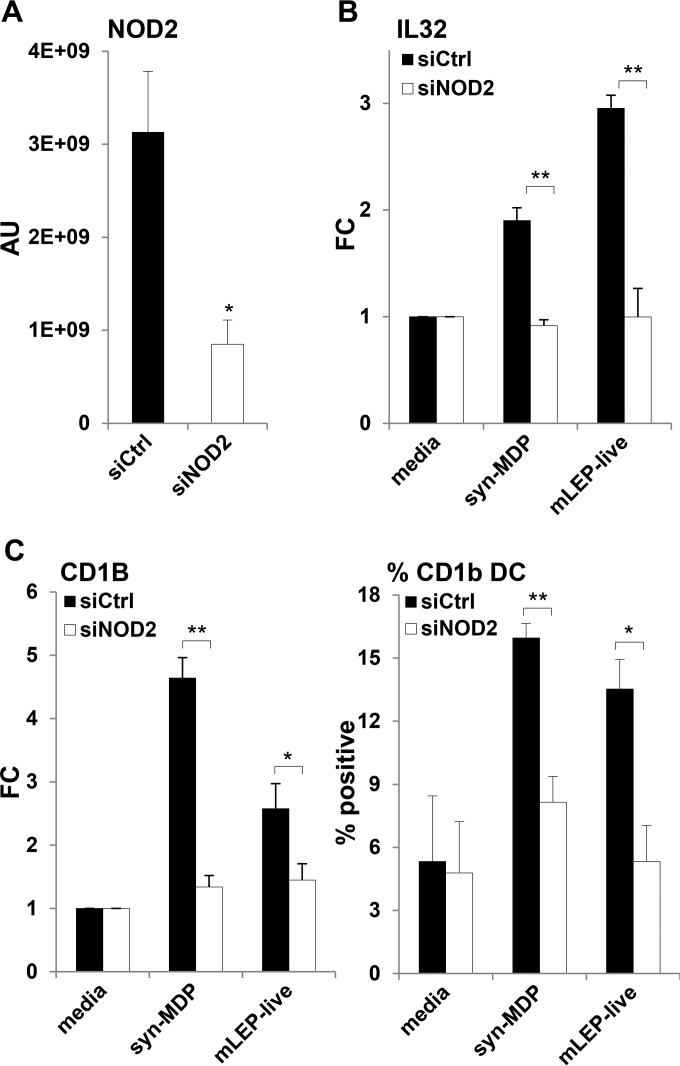
NOD2L is a potent inducer of IL-32 and DC differentiation. siRNA knockdown of NOD2 in purified human monocytes significantly reduced NOD2 expression, shown as arbitrary units (AU) (A), blocked syn-MDP and live M. leprae induction of IL-32 mRNA, shown as mean fold change (FC) (B), and reduced the induction of CD1b^+^ DC, shown as the percentage of positive cells (C). Data are represented as means ± standard errors of the means (SEM) (*n* = 4). Statistical significance was calculated by two-tailed Student's *t* test. Asterisks indicate statistically significant differences: *, *P* < 0.05; **, *P* < 0.01.

Although NOD2 single-nucleotide polymorphisms and NOD2 downstream immune responses are thought to contribute to the pathogenesis of leprosy, it is not clear whether M. leprae, which has a distinct MDP structure, activates the NOD2 pathway. To determine the role of NOD2 in leprosy infection, we silenced NOD2 gene expression in human monocytes using short interfering RNAs (siRNAs) (Fig. 1A) and measured the induction of IL-32 mRNA in response to infection with live M. leprae. IL-32 induction was measured, since it is specific to NOD2 versus TLR2/1 activation and is required to induce CD1b^+^ DC differentiation and cross-presentation, and its expression at the site of disease correlates with the self-limited versus progressive form of leprosy. Knockdown of NOD2 (siNOD2) almost completely blocked IL-32 induction in response to live M. leprae compared to the control (siCtrl)-treated cells (Fig. 1B). Similarly, knockdown of NOD2 blocked the response to the synthetic conventional MDP (*N*-acetylmuramyl-l-alanyl-d-isoglutamine, also called syn-MDP). At the same time, live M. leprae and syn-MDP induced expression of CD1b in monocytes, indicative of DC differentiation. Both live M. leprae and syn-MDP induced CD1b mRNA and protein expression, which was significantly reduced in the absence of NOD2 (Fig. 1C). These data indicate that NOD2 is crucial for the innate recognition of M. leprae in infected monocytes.

### Identification of the M. leprae ligand(s) that induces IL-32.

**FIG 2:**
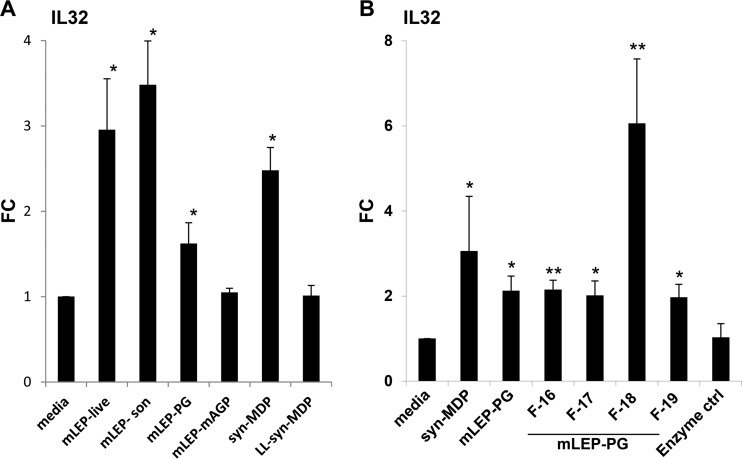
Induction of IL-32 by M. leprae. (A) Purified human monocytes were cultured with live M. leprae (MOI of 10) or 10 μg/ml of either sonicated bacilli (mLEP-son), digested M. leprae peptidoglycan (mLEP-PG), M. leprae mycolyl-arabinogalactan-peptidoglycan (mAGP), synthetic MDP (syn-MDP), or inactive synthetic MDP (LL-syn-MDP), and IL-32 gene expression was measured. (B) Four enriched fractions of muropeptides derived from mLEP-PG (mLEP-PG F-16, -17, -18, and -19) were tested for their ability to induce IL-32 expression and compared to digested M. leprae peptidoglycan (mLEP-PG) and synthetic MDP (syn-MDP). Data are represented as mean fold change (FC) compared to the medium control (ctrl) ± SEM (*n* = 6). Statistical significance was calculated by two-tailed Student's *t* test. Asterisks indicate statistically significant differences compared to media control: *, *P* < 0.05; **, *P* < 0.01.

To identify the M. leprae ligand(s) that regulates IL-32 expression, we cultured monocytes with live or sonicated bacilli, the M. leprae mycolyl-arabinogalactan-peptidoglycan (mAGP), the digested M. leprae peptidoglycan, or enriched fractions of muropeptides derived from M. leprae peptidoglycan. As reference controls, we compared the mycobacterial ligands to syn-MDP and inactive synthetic MDP (*N*-acetylmuramyl-l-alanyl-l-isoglutamine, or LL-syn-MDP) (Fig. 2A). The induction of IL-32 mRNA expression was significantly greater in cells treated with either live or sonicated M. leprae, the peptidoglycan fraction containing the muropeptides and syn-MDP, compared to the medium control (Fig. 2A). The M. leprae mAGP did not induce IL-32 expression. The mAGP complex is hydrophobic and insoluble and might not be accessible to host lytic enzymes that would release MDP, which is required for NOD2 activation. Additionally, M. leprae subcellular fractions, including cell wall core, cell wall protein, and cytosolic protein, which are expected to lack MDP, also did not induce IL-32 production (data not shown). The stereospecificity of NOD2 ligand recognition was confirmed in studies with LL-MDP which failed to induce IL-32.

The muropeptide fraction (M. leprae peptidoglycan) consisted of a heterogeneous mixture of muropeptides with potential modification sites on sugar and peptide residues. To identify the M. leprae ligand(s) within the M. leprae peptidoglycan preparation that was responsible for NOD2 activation, we further fractionated the digested M. leprae peptidoglycan by size exclusion chromatography (see Fig. S1 in the supplemental material) and tested these for their ability to induce IL-32 expression. The M. leprae peptidoglycan fraction 18 (F-18) was found to be the most potent, such that F-18 and the neighboring fractions were studied in greater detail. F-18 induced a 6-fold increase in IL-32 expression compared to the medium control (Fig. 2B). In comparison, F-16, F-17, and F-19 upregulated IL-32 expression by about 2-fold. The syn-MDP increased expression by 3-fold compared to that with medium alone.

F-18 of the M. leprae peptidoglycan digest and the two adjacent fractions (F-17 and F-19) were analyzed by LC-MS to determine their composition and relative quantity of muropeptides. Muropeptide composition was elucidated by interrogation of the MS data against a database of potential muropeptide structures and their calculated masses (Table 1). A comparison of the structures in the various M. leprae peptidoglycan fractions revealed that F-17 was comprised entirely of cross-linked muropeptides, while F-18 was a mixture of cross-linked and monomeric muropeptides and F-19 contained only monomeric muropeptides (Table 1). However, M. leprae peptidoglycan F-18 possessed a greater abundance of monomeric muropeptides in which the MurNAc residue was not modified to an anhydro form during enzymatic hydrolysis. Thus, this unmodified form of the monomeric muropeptide was likely the reason for the increased IL-32 induction by F-18.

**TABLE 1:** Muropeptide composition of the fractions tested^*a*^

Fraction no. and predicted muropeptide structure	Residue modification(s)		Observed mass^*d*^	Abundance (vol)
	Sugar^*b*^	Peptide^*c*^		
17				
Dimer (GlcNAc-MurNAc-Gly-d-Glu-DAP/GlcNAc-MurNAc-Gly-d-Glu-DAP)	Anhydro muramic acid	4 Amidation	1,668.7244	256,684
Dimer (GlcNAc-MurNAc-Gly-d-Glu-DAP-d-Ala/GlcNAc-MurNAc-Gly-d-Glu-DAP-d-Ala)	Anhydro muramic acid	4 Amidation	1,810.7952	272,907
Dimer (GlcNAc-MurNAc-Gly-d-Glu-DAP/GlcNAc-MurNAc-Gly-d-Glu-DAP)	Deacetylated, anhydro muramic acid	5 Amidation	1,625.7257	370,152
Dimer (GlcNAc-MurNAc-Gly-d-Glu-DAP/GlcNAc-MurNAc-Gly-d-Glu-DAP-d-Ala)	NA	4 Amidation	1,757.768	448,216
Dimer (GlcNAc-MurNAc-Gly-d-Glu-DAP/GlcNAc-MurNAc-Gly-d-Glu-DAP-d-Ala)	NA	4 Amidation	1,757.7705	523,833
Dimer (GlcNAc-MurNAc-Gly-d-Glu-DAP-d-Ala/GlcNAc-MurNAc-Gly-d-Glu-DAP-d-Ala)	NA	4 Amidation	1,828.8073	653,560
Dimer (GlcNAc-MurNAc-Gly-d-Glu-DAP/GlcNAc-MurNAc-Gly-d-Glu-DAP-d-Ala)	Anhydro muramic acid	4 Amidation	1,739.7597	804,009
Dimer (GlcNAc-MurNAc-Gly-d-Glu-DAP-d-Ala/GlcNAc-MurNAc-Gly-d-Glu-DAP-d-Ala)	Anhydro muramic acid	4 Amidation	1,810.7961	888,780
Dimer (GlcNAc-MurNAc-Gly-d-Glu-DAP-d-Ala/GlcNAc-MurNAc-Gly-d-Glu-DAP-d-Ala)	NA	4 Amidation	1,828.8062	1,293,773
Dimer (GlcNAc-MurNAc-Gly-d-Glu-DAP/GlcNAc-MurNAc-Gly-d-Glu-DAP-d-Ala)	Anhydro muramic acid	4 Amidation	1,739.7601	1,449,098
18				
Monomer (GlcNAc-MurNAc-Gly-d-Glu-DAP-d-Ala)^*e*^	NA	1 Amidation	924.3924	39,795
Dimer (GlcNAc-MurNAc-Gly-d-Glu-DAP-Gly/GlcNAc-MurNAc-Gly-d-Glu-DAP-d-Ala)	Deacetylated	3 Amidation	1,795.736^*f*^	11,361
Monomer (GlcNAc-MurNAc-Gly-d-Glu-DAP)^*e*^	Anhydro muramic acid	1 Amidation	835.3434	16,734
Dimer (GlcNAc-MurNAc-Gly-d-Glu-DAP-d-Ala/GlcNAc-MurNAc-Gly-d-Glu-DAP-d-Ala)	Deacetylated	3 Amidation, Gly	1,866.7642^*f*^	27,999
Dimer (GlcNAc-MurNAc-Gly-d-Glu-DAP-d-Ala/GlcNAc-MurNAc-Gly-d-Glu-DAP-d-Ala)	NA	4 Amidation	1,828.8115	17,990
Dimer (GlcNAc-MurNAc-Gly-d-Glu-DAP/GlcNAc-MurNAc-Gly-d-Glu-DAP-d-Ala)	Anhydro muramic acid	4 Amidation	1,739.763	17,117
Monomer (GlcNAc-MurNAc-Gly-d-Glu-DAP)^*e*^	NA	1 Amidation, Gly	932.3628^*f*^	15,261
Dimer (GlcNAc-MurNAc-Gly-d-Glu-DAP/GlcNAc-MurNAc-Gly-d-Glu-DAP)	Deacetylated	5 Amidation, methylation	1,657.7696	35,943
Dimer (GlcNAc-MurNAc-Gly-d-Glu-DAP/GlcNAc-MurNAc-Gly-d-Glu-DAP)	NA	2 Amidation, Gly	1,745.723	15,281
19				
Monomer (GlcNAc-MurNAc-Gly-d-Glu-DAP-d-Ala)^*e*^	Anhydro muramic acid	2 Amidation, Gly, methylation	976.4335	34,655
Monomer (GlcNAc-MurNAc-Gly-d-Glu-DAP-d-Ala)^*e*^	Anhydro muramic acid	1 Amidation	906.3814	200,361
Monomer (GlcNAc-MurNAc-Gly-d-Glu-DAP)^*e*^	Anhydro muramic acid	2 Amidation, Gly, methylation	905.3816	29,404
Monomer (GlcNAc-MurNAc-Gly-d-Glu-DAP)^*e*^	NA	1 Amidation	932.3689^*f*^	15,237

a The muropeptide fraction obtained from M. leprae peptidoglycan was further fractionated by size exclusion chromatography. The dominant muropeptides from each fraction were identified using LC-MS analysis by interrogating the MS data against a database search. The predicted structures, sugar residue modifications, peptide residue modifications, observed mass, and abundance are indicated for fractions 17 to 19.

b Anhydro muramic acid is a potential 1,6-anhydromuramic acid. Deacetylated indicates the loss of an N-acetyl group from one of the sugar residues. NA, not applicable.

c Amidation, amidation of carboxylic acid of d-Glu and/or DAP; Gly, d-Glu or DAP residues modified by a glycine residue; methylation, methylation of free carboxylic acid groups of d-Glu or DAP. The number (1 to 5) indicates the number of carboxylic acid residues that are amidated.

d All observed masses are H^+^ adducts unless otherwise noted.

e Uncross-linked muropeptide.

f Observed mass was a Na^+^ adduct.

### Innate immune responses to M. leprae MDP.

**FIG 3:**
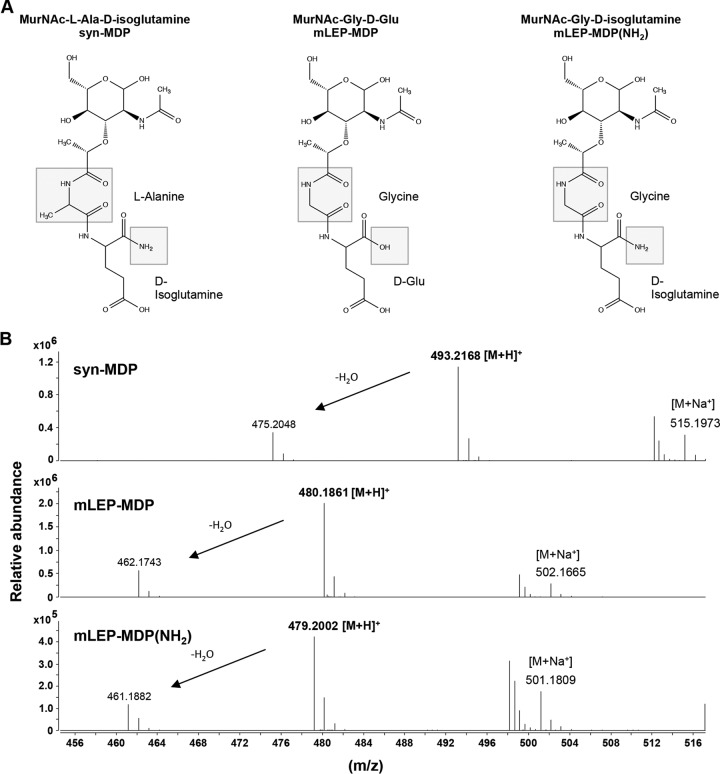
Chemical structures of synthetic MDP and M. leprae MDP. (A) Structural differences between the different MDPs are highlighted (box). The first amino acid residue of M. leprae MDP is Gly instead of l-Ala, and the d-Glu residue is amidated [mLEP-MDP(NH_2_)] or nonamidated (mLEP-MDP). (B) mLEP-MDP(NH_2_) and mLEP-MDP were synthesized enzymatically, and the molecular structures were confirmed by mass spectrometry: [M+H]^+^ of 493.2168 *m/z* belongs to syn-MDP, [M+H]^+^ of 480.1861 *m/z* has a calculated molecular formula of C_18_H_29_N_3_O_12_, which is consistent with the molecular formula of mLEP-MDP, and [M+H]^+^ of 479.2002 *m/z* has a calculated molecular formula of C_18_H_30_N_4_O_11_, which is also the molecular formula of mLEP-MDP(NH_2_).

The structure of M. leprae peptidoglycan-derived muramyl dipeptide is unique, as the first amino acid residue of the tetrapeptide side chain is Gly instead of l-Ala and the d-Glu residues are not fully amidated. Thus, MDP naturally derived from M. leprae would contain a mixture of amidated and nonamidated *N*-acetylmuramyl-glycinyl-d-isoglutamine (Fig. 3A). To further test structure-function relationships of M. leprae MDP, we enzymatically synthesized the amidated and nonamidated form of MDP and confirmed the molecular structures by mass spectrometry (Fig. 3B; see also Fig. S1 in the supplemental material). The [M+H]^+^ molecular ion of *m/z* 493.2168 belonged to the syn-MDP standard. The [M+H]^+^ molecular ion of *m/z* 480.1861 yielded a calculated molecular formula of C_18_H_29_N_3_O_12_ that was consistent with the molecular formula of M. leprae MDP, and the [M+H]^+^ molecular ion of *m/z* 479.2002 represented amidated MDP of M. leprae MDP(NH_2_) with a calculated molecular formula of C_18_H_30_N_4_O_11_ (Fig. 3B; see also Fig. S1).

**FIG 4:**
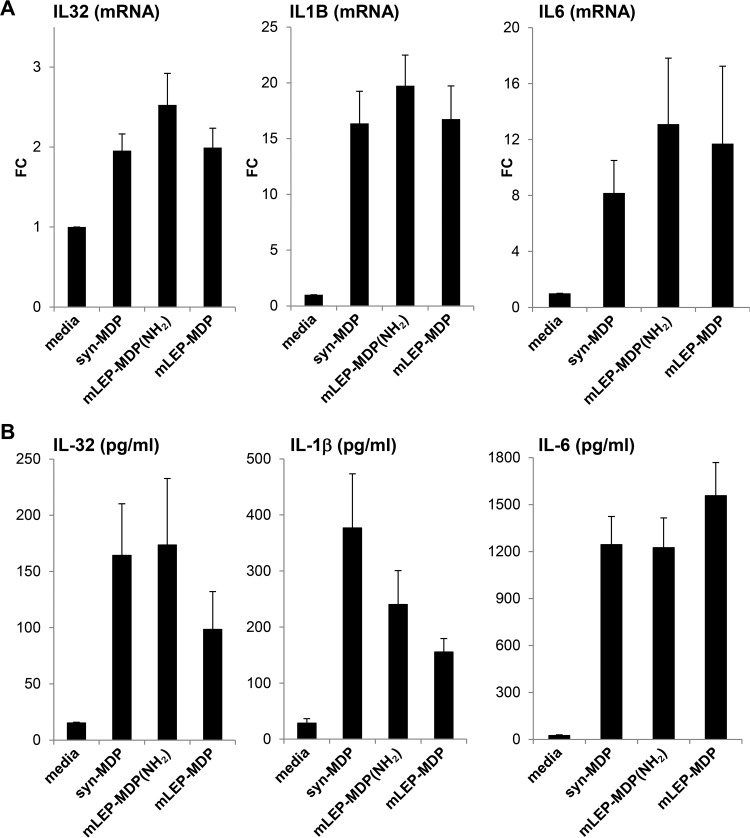
Cytokine response to structurally distinct MDPs. Purified human monocytes were activated by adding 1 μg/ml of syn-MDP, mLEP-MDP(NH_2_), or mLEP-MDP. After 24 h, IL-32, IL-1β, and IL-6 induction was measured by mRNA expression as fold change (FC) compared to the medium control (A) and protein secretion (B). Data are represented as means ± SEM (*n* ≥ 6).

The cytokine induction in human monocytes was compared for these structurally distinct MDPs. The proinflammatory cytokines IL-32, IL-1β, and IL-6 were all significantly induced by syn-MDP and the amidated and nonamidated M. leprae MDP, as measured by mRNA expression (Fig. 4A) and protein secretion (Fig. 4B), indicating that the unique structure of the M. leprae MDP does not interfere with its ability to activate an innate immune response. The amidated form of the M. leprae MDP showed a trend, albeit not significant, to induce a stronger IL-32, IL-1B, and IL-6 mRNA response. IL-1B mRNA induction was 1.2-fold higher in monocytes stimulated with the amidated form of the M. leprae MDP than the nonamidated and synthetic form. However, the level of secreted IL-1β protein was 1.6-fold higher in monocytes cultured with the syn-MDP than the amidated M. leprae MDP and 2.4-fold higher than the nonamidated form of M. leprae MDP. The discrepancy between the induction of IL-1B mRNA and protein could be a consequence of differences in inflammasome activation, which requires further investigation of caspase/inflammasome components. To determine whether these compounds are comparably potent throughout a wide range of concentrations, we performed a dose titration and measured IL-32 induction. The compounds induced comparable amounts of IL-32 throughout a range of concentrations, with peak induction at 1 μg/ml (see Fig. S2 in the supplemental material).

### M. leprae MDP activates NOD2 and induces human monocytes to differentiate into DC.

**FIG 5:**
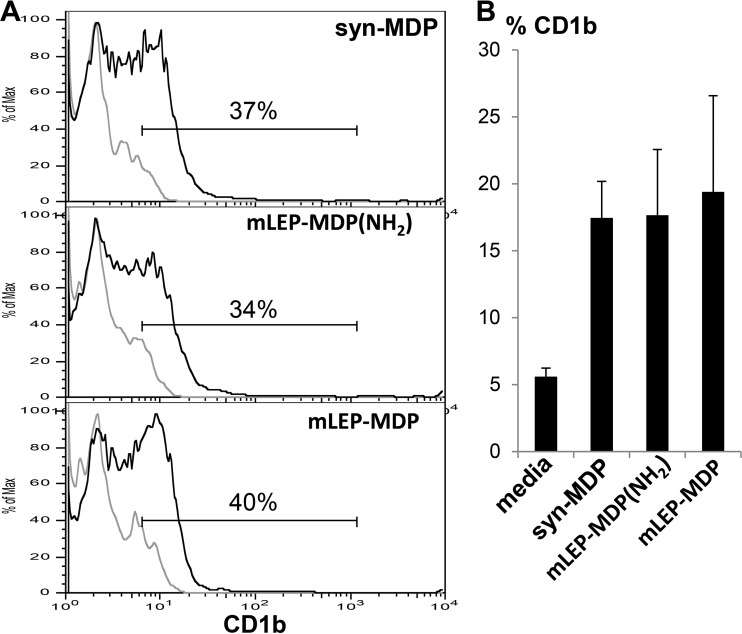
Induction of DC differentiation by different MDPs. Purified human monocytes were activated using syn-MDP, mLEP-MDP(NH_2_), and mLEP-MDP, and DC differentiation was measured by flow cytometry for CD1b expression. A representative histogram (A) and the mean percentage of CD1b-positive cells ± SEM (B) are shown (*n* = 5).

CD1b^+^ DC are potent antigen-presenting cells in induction of an adaptive T cell response in leprosy (28, 29). Here, we found that M. leprae MDP induces human monocytes to differentiate into CD1b^+^ DC with a magnitude similar to that of syn-MDP (Fig. 5). The frequency of CD1b^+^ DC at the site of disease correlates with clinical forms of the disease, i.e., greater in T-lep than in L-lep lesions (30). In addition, we showed that gene expression of both IL-32 and CD1B were significantly greater in T-lep than L-lep lesions (3). Therefore, the ability of M. leprae MDP to induce both IL-32 and CD1b^+^ DC is linked to host defense at the site of infection.

### M. leprae MDP is recognized via NOD2.

**FIG 6:**
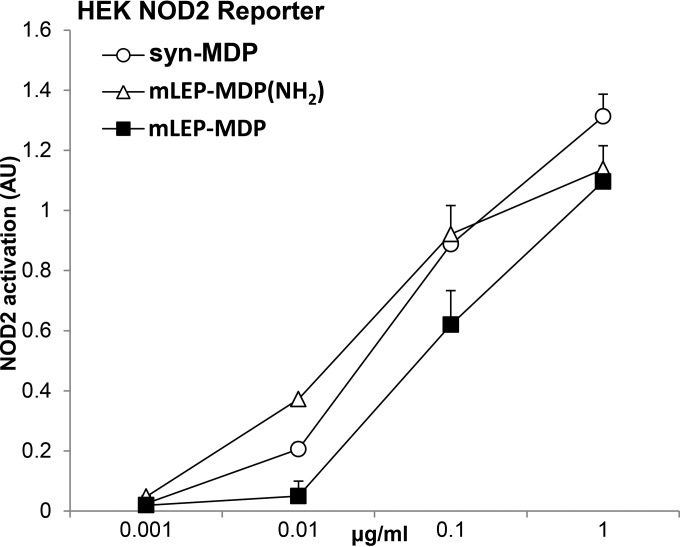
Quantification of NOD2 signaling induced by structurally distinct MDPs. HEK-NOD2 reporter cells were used to quantitatively assess NOD2 activation by stimulating cells with structurally different MDP compounds [syn-MDP, mLEP-MDP-(NH_2_), and mLEP-MDP] over a range of concentrations (0.001 to 1 μg/ml). Data are represented as mean arbitrary units (AU) ± SEM (*n* = 6).

To demonstrate that recognition of the M. leprae MDP was mediated by NOD2, we used a HEK NOD2 reporter cell line. This allows us to quantitatively assess NOD2 activation by structurally different compounds over a range of concentrations. At lower concentrations (0.01 and 0.1 μg/ml), the amidated form of M. leprae MDP was found to be a more potent activator of NOD2; however, this difference was not observed at higher concentrations (1 μg/ml) (Fig. 6). These data show that the M. leprae MDP is recognized by NOD2 in transfected HEK reporter cells.

**FIG 7:**
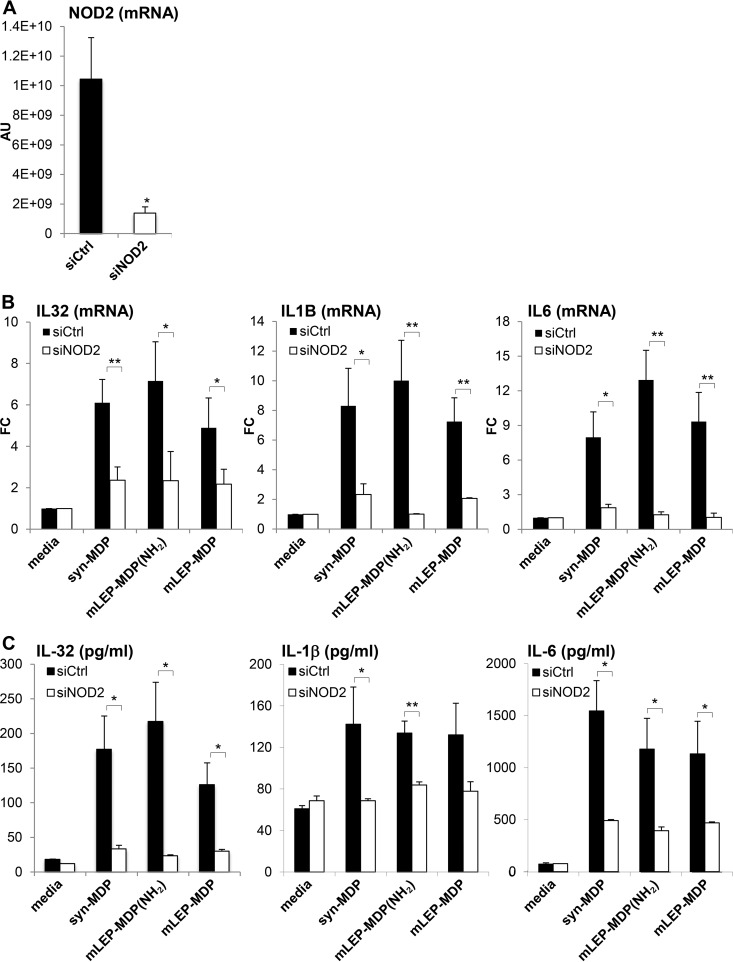
siNOD2 abolished MDP-induced cytokine response. (A) Purified human monocytes were transfected with siNOD2 or siCtrl, and NOD2 gene expression was measured and is shown in arbitrary units (AU). siNOD2 knockdown monocytes were stimulated with 1 μg/ml of syn-MDP, mLEP-MDP(NH_2_), or mLEP-MDP and induction of IL-32, IL1B, and IL-6 mRNA, shown as fold change (FC) compared to the medium control (B), and protein expression was measured (C). Data are represented as means ± SEM (*n* = 4). Statistical significance was calculated by two-tailed Student's *t* test. Asterisks indicate statistically significant differences compared to the medium control: *, *P* < 0.05; **, *P* < 0.01.

The role of NOD2 in M. leprae MDP-induced immune responses was tested by knockdown of NOD2 expression in monocytes using siRNAs with the subsequent measurement of cytokine responses. NOD2 mRNA expression was inhibited in monocytes transfected with siNOD2 by about 90% compared to that in siCtrl-transfected cells (Fig. 7A). The induction of IL-32, IL-1B, and IL-6 mRNAs (Fig. 7B) and protein (Fig. 7B) was significantly reduced by the knockdown of NOD2 mRNAs for all forms of MDP tested. Together, these data indicate that M. leprae MDP, despite its unique structure, activates human monocytes via NOD2 to trigger a range of innate immune responses with relevance to the pathogenesis of leprosy which are comparable to those induced by the MDP structures found in most other bacteria.

## DISCUSSION

The importance of NOD2 activation in immune responses has become evident since the identification of MDP as a key component of mycobacterial cell walls responsible for conferring adjuvant activity (6, 31) in inducing both B cell (32) and T cell (33) responses. NOD2 recognition of MDP triggers the induction of specific inflammatory responses to combat bacterial infection, including the production of IL-32, which in leprosy is linked to host defense against the pathogen M. leprae. Nevertheless, it was unknown whether the unique structure of the M. leprae MDP activates a NOD2 response. Here, we demonstrate that infection of monocytes with M. leprae induces the NOD2-dependent production of IL-32 as well as DC differentiation. The M. leprae MDP, which includes a replacement of l-Ala with Gly in the peptide side chain with or without amidation of the d-Glu residue, triggered IL-32 production and DC differentiation. These data provide evidence that host pattern receptors of the innate immune system can recognize naturally occurring structural variants of MDP, including the M. leprae MDP.

Investigation of the effect of the structure of the M. leprae MDP on innate immune responses was undertaken to understand the relationship between structure and function of this microbial ligand. Our strategy was to measure induction of IL-32 in monocytes, given that the M. leprae peptidoglycan fraction containing the muropeptides uniquely triggers IL-32. There are several modifications of the M. leprae MDP structure that could relate to bioactivity. First, the replacement of l-Ala with Gly in the peptide side chain of the M. leprae MDP did not alter NOD2 activation. Second, the presence or absence of an amide in the d-Glu residue in combination with Gly did not affect the innate immune response. Third, we were able to confirm that stereospecific alterations block NOD2 activity, as d-isoglutamine-to-l-isoglutamine alteration renders MDP inactive (8). Fourth, the muramic acid of M. leprae MDP is not *N*-glycolylated, as this bacterium does not contain a functional *namH* gene but appeared to be potent in stimulating cytokine responses and DC differentiation (34). Previously, study of the *namH* mutant of M. tuberculosis suggested that *N*-glycolylation of muramic acid was required to maximally induce TNF-α and IL-6 in macrophages; however, these particular cytokine responses required costimulation from LPS or trehalose dimycolate (15). Alternatively, other substitutions might compensate for the lack of *N*-glycolylation (14). We note that the muramic acid of M. leprae is *N*-acetylated, unlike other mycobacterial MDPs. In summary, the unique structure of the M. leprae MDP does not interfere with its ability to activate the innate immunity *in vitro*. Based on our studies indicating that live M. leprae induces monocytes to release IL-32 and to differentiate into CD1b^+^ DC, and that NOD2 downstream immune responses, including IL-32, are expressed at the site of leprosy infection, it is reasonable to speculate that the unique structure of M. leprae MDP does not significantly alter immune activation *in vivo*. In addition to inducing IL-32, M. leprae MDP stimulated monocyte release of IL-1β and IL-6, as well as of TNF-α and IL-12p40 (data not shown), and induced DC differentiation.

The ability of the M. leprae MDP to induce proinflammatory cytokines as well as DC differentiation most likely contributes to host defense, given that NOD2, IL-32, and CD1^+^ DC are more frequent in the self-limited T-lep versus progressive L-lep lesions (3). Consistent with this hypothesis, single-nucleotide polymorphisms in the NOD2 gene have implicated this PRR as the key innate immune receptor that contributes to host defense in leprosy (4, 5). A number of studies have implicated *NOD2* directly in other mycobacterial diseases. In tuberculosis (TB), nonsynonymous variants of *NOD2* were shown to be associated with active disease in a cohort of patients from Houston (35). Furthermore, detection of M. tuberculosis and M. paratuberculosis in human and murine macrophages is *NOD2* dependent (36, 37), and *NOD2* is important for the cytokine response and NO production in mouse macrophages infected with M. tuberculosis (38, 39). In a mouse model of M. tuberculosis infection *NOD2* deficiency did not affect the early phase of infection and bacterial burden (39), but the pulmonary bacterial burden was increased and survival decreased in NOD2-deficient mice (38). In addition to leprosy, human variants of *NOD2* have been associated with susceptibility to Crohn's disease (4, 5, 40, 41). It is interesting that Crohn's disease is a chronic granulomatous disorder of the gut with similarities to Johnne's disease, a mycobacterially induced granulomatous disorder in cows. However, an association of Crohn's disease with mycobacterial infection remains controversial. It should be noted that despite years of intensive research, the mechanism by which single-nucleotide polymorphisms (SNPs) in *NOD2* lead to the enhanced inflammation associated with Crohn's disease remains enigmatic (4, 5, 42).

It is unclear why M. leprae has evolved to possess a structural variant of MDP, except to possibly escape immune detection (10). However, our data indicate that NOD2 recognizes these naturally occurring structural variants of MDP to mount an effective host response. Future studies using patient monocytes and/or transfected cell lines are required to determine whether mutations in NOD2 lead to altered responses against certain structural variants of MDP, contributing to the pathogenesis of leprosy infection.

## Supplementary Material

Supplemental material
