# Supplementary material for: Human NOD2 Recognizes Structurally Unique Muramyl Dipeptides from Mycobacterium leprae
Source: Infect Immun. 2016 Aug 19;84(9):2429–38. doi: 10.1128/IAI.00334-16 (PMC4995902; doi:10.1128/IAI.00334-16)
Supplement: Supplemental material [file supp_84_9_2429__index.html]

Supplemental material 

# Human NOD2 Recognizes Structurally Unique Muramyl Dipeptides from Mycobacterium leprae

## Supplemental material

- Supplemental file 1 -

  Fig. S1. Preparation of natural and synthetic NOD2 ligands of *M. leprae*. Fig. S2. Titration of structurally different MDP for IL-32 induction.

  PDF, 175K
