# Supplementary material for: Human NOD2 Recognizes Structurally Unique Muramyl Dipeptides from Mycobacterium leprae
Source: Infect Immun. 2016 Aug 19;84(9):2429–38. doi: 10.1128/IAI.00334-16 (PMC4995902; doi:10.1128/IAI.00334-16)
Supplement: Supplemental material [file IAI.00334-16_zii999091788so1.pdf]

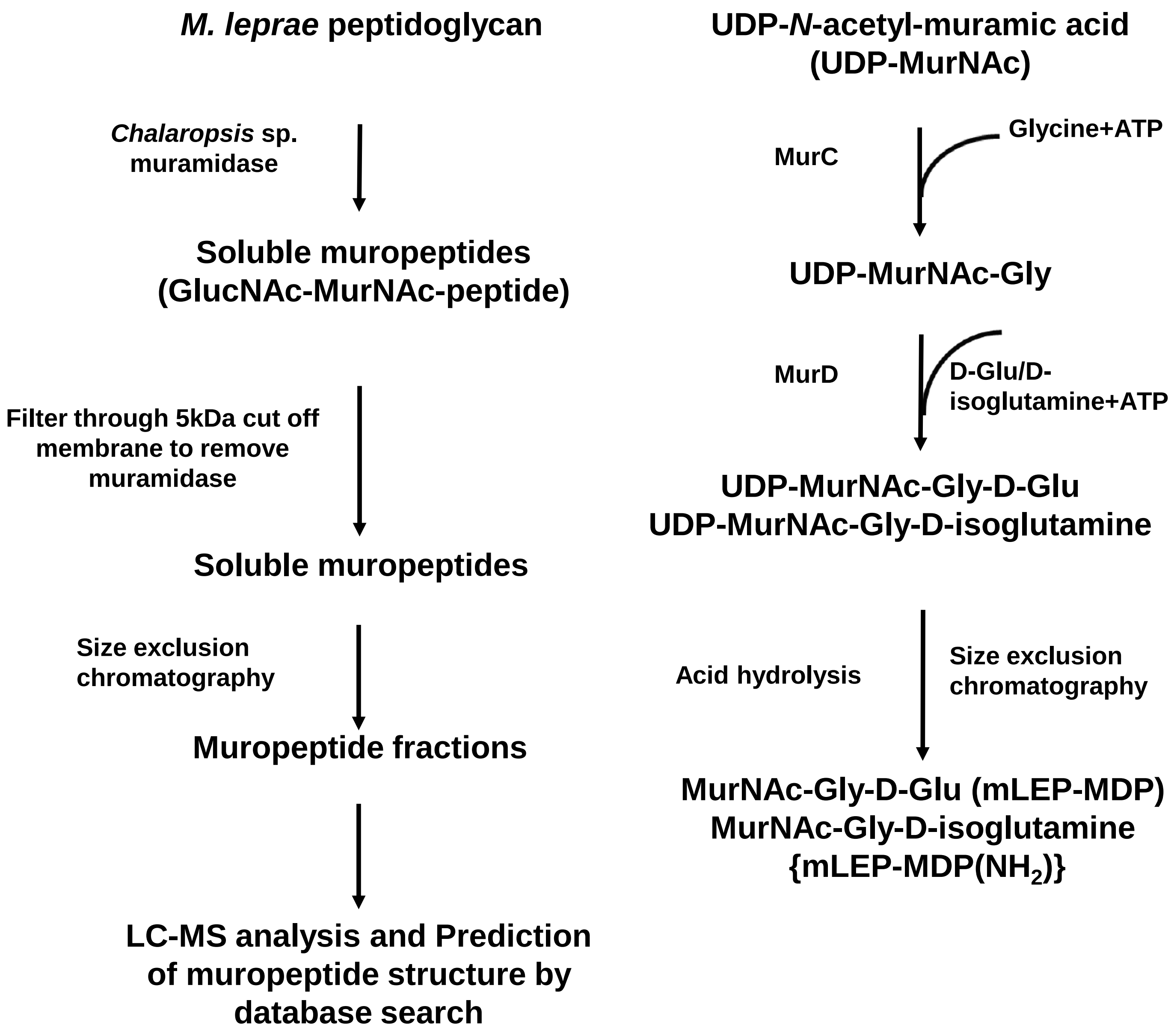

FIG S1 Preparation of natural and synthetic NOD2 ligands of *M. leprae.* A flowchart showing the steps to prepare the muropeptide fractions from *M. leprae* and the synthetic MDP variants (A). Amino acid composition analysis of syn-MDP, mLEP-MDP and mLEP-MDP(NH_2) by GC-MS (B).

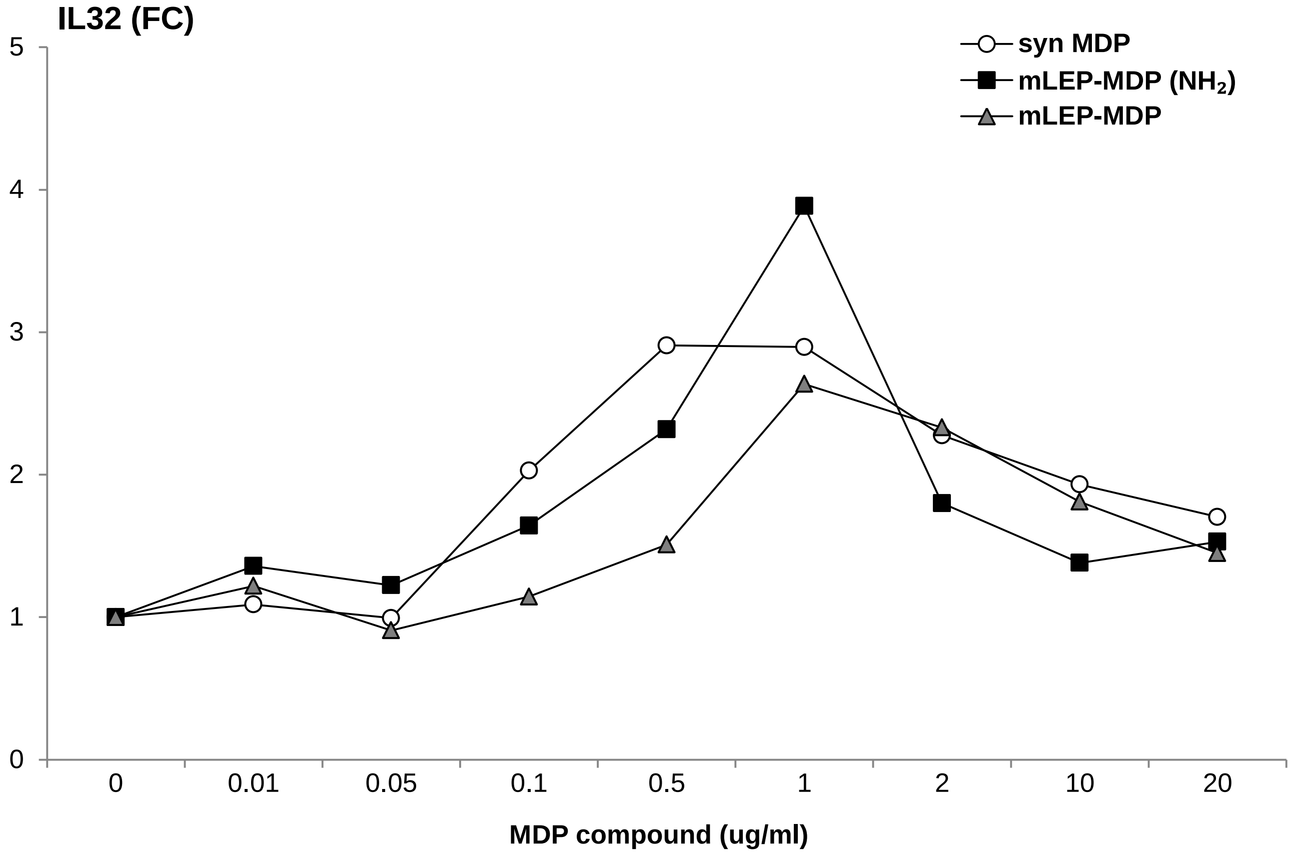

FIG S2 Titration of structural different MDP for IL32 induction. Purified human monocytes were activated using syn-MDP, mLEP-MDP(NH_2) and mLEP-MDP and IL32 gene induction was quantified by PCR. A representative histogram is shown.
